# Regulatory T cell-derived extracellular vesicles modify dendritic cell function

**DOI:** 10.1038/s41598-018-24531-8

**Published:** 2018-04-17

**Authors:** Sim L. Tung, Dominic A. Boardman, Monica Sen, Marilena Letizia, Qi Peng, Nicole Cianci, Laura Dioni, Leo M. Carlin, Robert Lechler, Valentina Bollati, Giovanna Lombardi, Lesley A. Smyth

**Affiliations:** 1 Medical Research Council (MRC) Centre for Transplantation, King’s College London, London, UK; 2 National Institute for Health Research (NIHR) Comprehensive Biomedical Research Centre, Guy’s and St. Thomas’ NHS Foundation Trust and King’s College London, London, UK; 3 EPIGET LAB, Department of Clinical Sciences and Community Health, Università degli Studi di Milano, Milan, Italy; 4 Cancer Research UK, Beatson Institute, Glasgow, UK; 5 School of Health, Sport and Bioscience, University of East London, London, UK

## Abstract

Regulatory T cells (Treg) are a subpopulation of T cells that maintain tolerance to self and limit other immune responses. They achieve this through different mechanisms including the release of extracellular vesicles (EVs) such as exosomes as shown by us, and others. One of the ways that Treg derived EVs inhibit target cells such as effector T cells is via the transfer of miRNA. Another key target for the immunoregulatory function of Tregs is the dendritic cells (DCs). In this study we demonstrate directly, and for the first time, that miRNAs are transferred from Tregs to DCs via Treg derived EVs. In particular two miRNAs, namely miR-150-5p and miR-142-3p, were increased in DCs following their interaction with Tregs and Treg derived exosomes. One of the consequences for DCs following the acquisition of miRNAs contained in Treg derived EVs was the induction of a tolerogenic phenotype in these cells, with increased IL-10 and decreased IL-6 production being observed following LPS stimulation. Altogether our findings provide data to support the idea that intercellular transfer of miRNAs via EVs may be a novel mechanism by which Tregs regulate DC function and could represent a mechanism to inhibit immune reactions in tissues.

## Introduction

Naturally occurring, thymus derived CD4^+^CD25^+^FoxP3^+^ regulatory T cells (Treg) play a fundamental role in the maintenance of immunological tolerance, preventing autoimmunity, as well as limiting immune responses^1–3^. Although they can act directly on effector T cells (Teffs), Tregs also target the orchestrators of the immune response, the dendritic cells (DCs)^4–7^. Recently, Yan *et al*. have shown, using intravital microscopy, that endogenous murine Tregs form prolonged contacts with injected LPS-treated DCs in lymph nodes, which was enhanced in the presence of interleukin (IL)−2^8^. These authors also measured the interaction times between antigen specific Teffs and DCs in the absence or presence of IL-2 pre-treated polyclonal Tregs. They found that DC-Teff cell contact time was significantly reduced in the presence of Tregs^8^. This *in vivo* data confirmed previous *in vitro* observations^6,7^ whereby Tregs, not treated with IL-2, clustered around DCs inhibiting Teff-DC interactions. The molecular bases of DC-Treg interactions was also recently revisited by Chen *et al*., using super resolution imaging^9^. These authors showed that the interaction between these two cell types was dependent on strong LFA-1-ICAM-1 binding by Tregs, as previously described^6^. This altered the cytoskeleton of DCs leading to sequestering of a molecule involved in the immunological synapse (IS), called Fascin 1, to the contact area between Treg and DC. In doing so, Fascin1 availability for IS formation between Teffs and DCs was reduced^9^.

This is just one way by which Tregs have been found to modify DCs^5^. Several mechanisms, IDO production, CD39/CD73 expression induced adenosine production and CTLA-4-facilitated removal of co-stimulatory molecules, CD80 and CD86, from the membrane of the DCs^10,11^ have all been attributed to how Treg modify DCs leading to reduced T cell responses. Regardless of the mechanism used, exposure to Tregs alters the ability of DCs to activate T cells and modifies cytokine profile^8,12–16^. In several *in vitro* studies Treg-treated DCs displayed reduced expression of CD80 and CD86^6^ as well as CD38 and CD83, in the human setting, compared to immature and mature DCs^17^. In addition they release inhibitory cytokines including IL-10, transforming growth factor (TGF)-β and IL-35 and have reduced IL-12 production following TLR activation^5,17^.

We, and others, have described the release of immune inhibitory exosomes from murine Tregs^18–20^. Given that the isolation protocols used to isolate exosomes in these studies may also include other small vesicles, as described by Kowal *et al*. the term extracellular vesicles (EVs) will be used in this manuscript instead of exosomes^21^. These structures have been shown to facilitate the intercellular transfer of small non-coding RNAs, microRNAs (miRNAs), including Let 7b, Let 7d, miR-150 and miR-155 from Tregs to Teffs^18,19^. Importantly miRNAs in Treg EVs were functional following transfer, repressing target mRNAs in the Teffs both *in vitro* and *in vivo*^19^. Whether miRNAs are transferred by Treg EVs to DCs has yet to be addressed, however Okoye *et al*. observed that fluorescent oligonucleotide duplexes (FL-dsRNA) expressed in murine Tregs could be acquired by DCs following co-culture suggesting that the transfer of miRNAs between these cells is a possibility^19^. In addition, the intercellular transfer of miRNAs containing EVs from murine T cells to APCs via vesicles has been observed^22^. Recently, CD8 suppressor murine T cells were found to release EVs containing miR-150, which modified macrophage function^18,23^.

A better understanding of the contribution of Treg derived EVs to maintaining tolerance is warranted. Given that DCs and Tregs interact *in vivo* and that the miRNAs expressed in DCs play a key role in modulating their function, specifically their cytokine production and their phagocytic capacities^24^, we investigated whether miRNAs present in Treg derived EVs are acquired by DCs and whether this affects the function of these cells.

## Results

### Murine Tregs and their EVs modulate DC cytokine production

**Figure 1:**
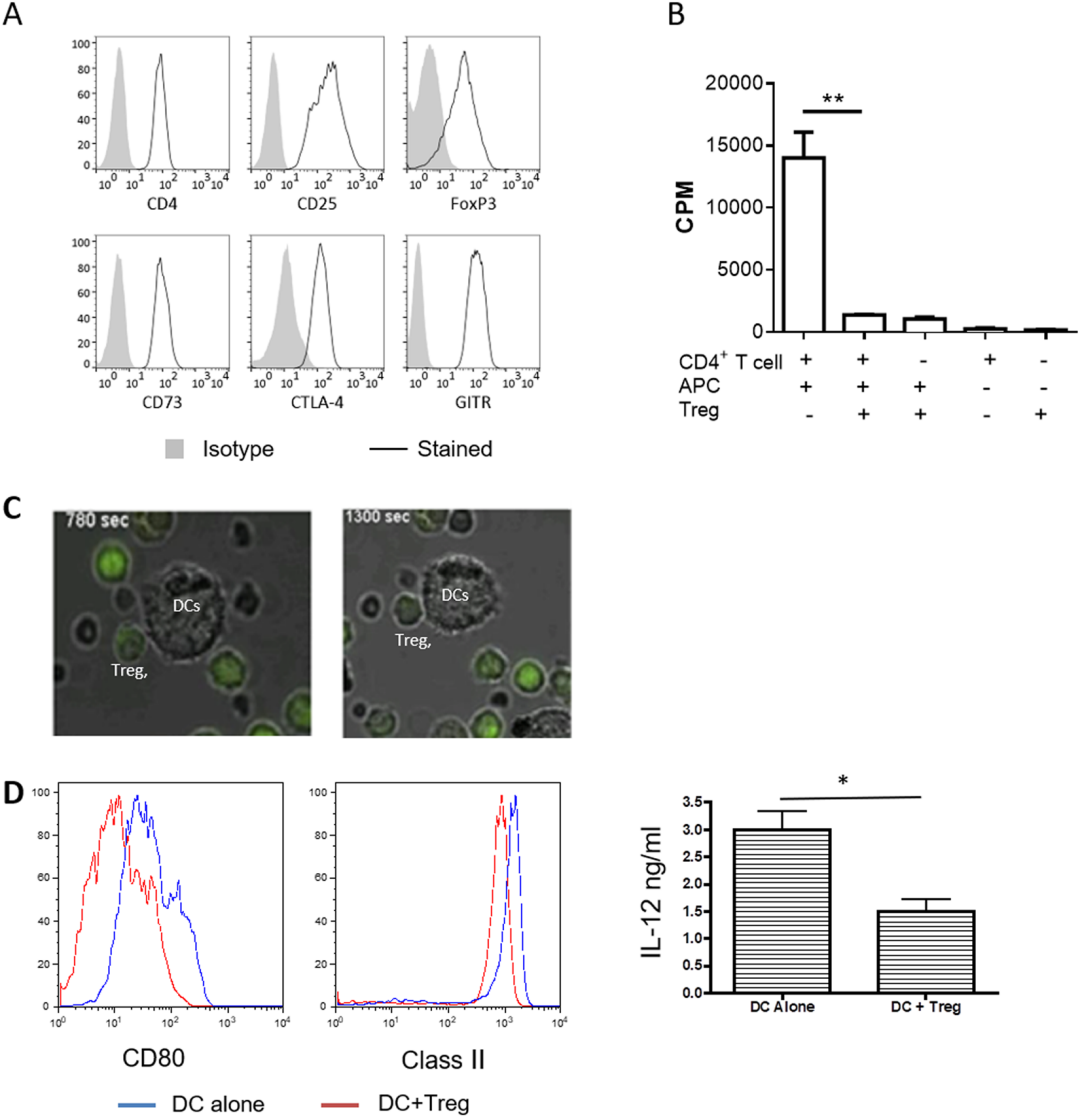
Murine CD4^+^CD25^+^ Tregs induce a tolerogenic phenotype in dendritic cells following co-culture. (**A**) Flow cytometry plots showing the expression of typical Treg markers by CD4^+^CD25^+^ T cells from a previously established cell line (black line). Isotype control staining is shown in solid grey. Data shown is representative of 8 experiments. (**B)** Suppressive capacity of CD4^+^CD25^+^ dTregs. B6 CD4^+^ T cells were stimulated with BALB/c APCs in the presence or absence of an equal number of dTregs. Co-cultures were performed for 3 days and proliferation was measured by ^3^H-thymidine incorporation. Data are representative of 5 experiments. (**C**) Confocal microscopy image of DCs co-cultured with CD4^+^CD25^+^ dTregs (green). Data are representative of 2 experiments. **(D)** Activation capacity of BM-DCs following co-culture with dTregs. BALB/c BM-DCs were co-cultured alone (blue line) or with dTregs (red line) for 24 hours to induce a tolerogenic phenotype. CD11c^+^CD4^−^ DCs were isolated by cell sorting and activated with 100 ng/mL LPS overnight. MHC class II (I-A^d^) and CD80 expression was measured by flow cytometry and IL-12 (ng/ml) was measured by cytokine-specific ELISA. Data represents 3 individual experiments that were performed in duplicates. Statistical significance was determined using a two-tailed paired Student’s t-test where *p < 0.05 and **p < 0.01. CPM = counts per minute.

DCs are a major target of CD4^+^CD25^+^Foxp3^+^ Tregs. One of the consequences of this cellular interaction is the acquisition by DCs of a ‘tolerogenic’ phenotype^5,6^. The hypothesis that was tested in this study is whether EVs released by Tregs contribute to the ‘tolerogenic’ effect of DCs. To test this possibility a very well characterised murine Treg line, generated by stimulating B6 CD4^+^CD25^+^Foxp3^+^ cells *in vitro* with allogenic BALB/c DCs was used. This Treg line has direct allospecificity for BALB/c MHC class II molecule, I-A^d^, antigens^25–27^ and will be referred to as dTregs throughout the manuscript. Phenotypic analysis of the BALB/c-specific Tregs showed that they expressed CD4, the IL-2 receptor α-chain (CD25) and the lineage-defining transcription factor FoxP3, as well as CTLA-4, CD73 and GITR (Fig. 1A). These cells were also highly suppressive; proliferation of B6 CD4^+^ responder T cells (Tresp) upon stimulation with allogeneic BALB/c APCs was significantly decreased (p = 0.0092) by the presence of dTregs at a 1:1 Treg:Tresp ratio (Fig. 1B). In addition, these Tregs interacted with BALB/c BM-DCs. Real time imaging of BALB/c DCs co-cultured with CFDA (green) labelled BALB/c-specific Tregs indicated that dTregs formed conjugates with BALB/c derived bone-marrow (BM)-DCs (Fig. 1C). Before evaluating the effect of Treg derived EVs, we confirmed that by co-culturing Tregs with BM-DCs, the expression of CD80 and MHC class II on DCs was reduced as well as the production of the pro-inflammatory cytokine IL-12 induced by LPS-mediated activation. These results confirmed previous findings that dTregs can modify DC phenotype and function (Fig. 1D).

**Figure 2:**
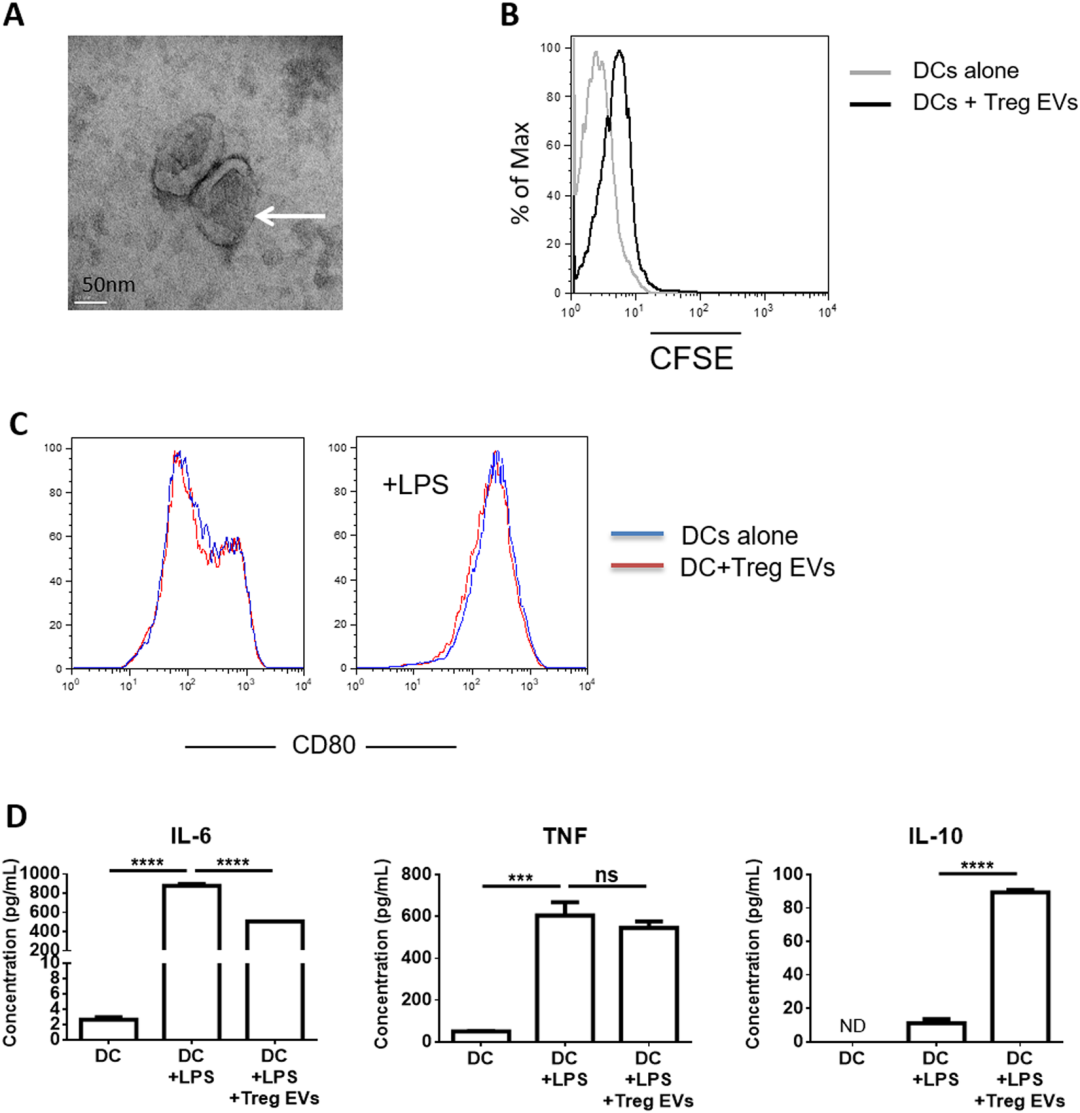
Murine Treg derived EVs are acquired by dendritic cells and alter cytokine production. (**A)** Electron micrograph image of EVs released from dTregs activated by plate-bound αCD3/CD28 antibodies isolated by ultracentrifugation. Scale bar indicates 50 nm. Representative image from 3 experiments is shown. (**B)** Treg cells were labelled with CFSE and activated by plate bound αCD3/CD28 antibodies for 24 hours. Supernatant was collected, cells and cell debris depleted and EVs isolated via ultracentrifugation (Treg EVs). BM-DCs were cultured alone or co-cultured with CFSE^+^ Treg EVs for 24 hours. Flow cytometry histogram plot showing CFSE expression levels of DCs cultured alone (grey) and DCs co-cultured with CFSE^+^ Treg EVs (black). Data is representative of 3 independent experiments. (**C**) BM-DCs were co-cultured alone (blue line) or with Treg derived EVs (red line) in the presence of 100 ng/mL LPS and CD80 expression was measured by flow cytometry after 24 hours. (**D**) IL-6, TNF and IL-10 cytokine production by BM-DC (DC+LPS), BM-DCs pre-treated with Treg EVs for 24 hours (DC + LPS + Treg EVs) and untreated BM-DCs (DCs) following LPS activated (24 hours) was assessed by cytometric bead array and flow cytometry. Data represents 2 independent experiments that were performed in technical triplicates, mean + SEM is shown. Statistical significance was determined using One-way ANOVA and Tukey’s multiple comparison test. ***p < 0.001, ****p < 0.0001, NS = not significant and ND = not determined.

Next we assessed whether dTreg derived EVs modified DCs in a similar manner. As expected, Tregs produced EVs of around 100 nm following TCR activation (Fig. 2A and Supplemental Fig. 3) which were found to be ‘acquired’ by BM-DCs upon co-culture (Fig. 2B). In contrast to Tregs, dTreg derived EVs did not influence the expression of the co-stimulatory molecule CD80 (Fig. 2C) by BM-DCs. However, we did observe that following LPS activation, IL-6, but not TNFα, was significantly reduced in Treg derived EVs treated BM-DCs, as compared to controls (Fig. 2C). Interestingly, we also observed that IL-10 release was significantly increased (Fig. 2C) in LPS activated DCs that had been co-cultured in the presence of Treg-derived EVs but no changes were observed for TNFα. This was not due to the presence of IL-10 in the EVs (data not shown).

Taken together, our data suggests that both dTregs and their EVs modify DCs following interaction with the latter, predominantly affecting cytokine production, although EVs and Tregs affect BM-DCs in a different manner.

### miRNAs present in Tregs were increased in BM-DCs following co-culture

**Figure 3:**
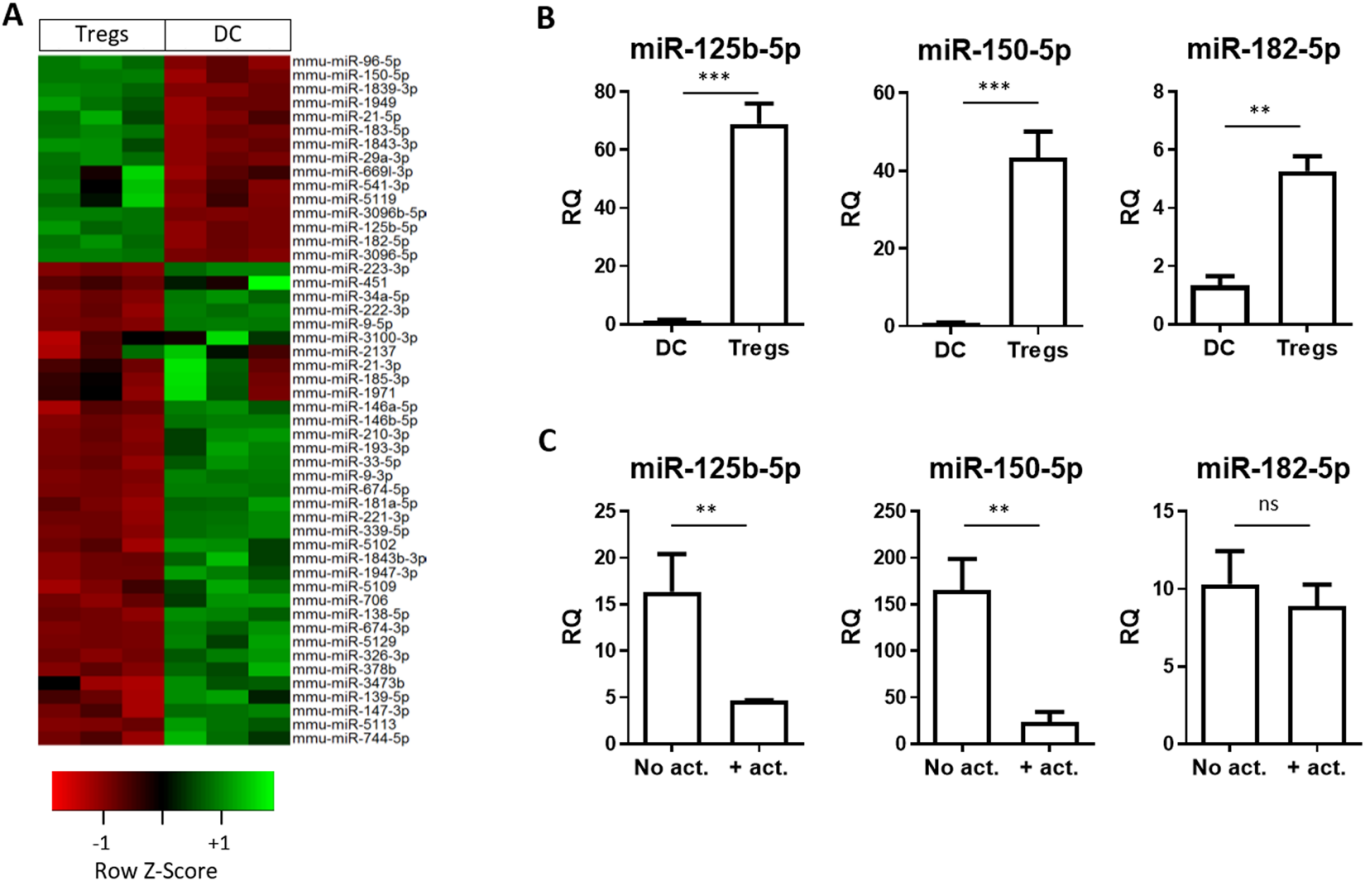
Murine CD4^+^CD25^+^ dTregs express high levels of specific miRNAs compared to CD11c^+^ BM-DCs. (**A**) Microarray heat map diagram showing a two-way unsupervised hierarchical clustering of miRNAs by CD4^+^CD25^+^ dTregs and BM-DCs microRNA. (**B)** Bar graphs (mean+SEM) showing the relative quantity (RQ) of miR-125b-5p, miR-150-5p, and miR-182-5p in BM-DCs and dTregs, as measured by qPCR and normalised relative to RNU6-2 and SCARNA17. Data represents 2 individual experiments that were performed in duplicates. Statistical significance was determined using a two-tailed paired Student’s t-test where **p < 0.01 and ***p < 0.001. (**C**) Bar graphs (mean + SEM) showing the RQ of indicated miRNAs by Tregs immediately before (no act) and after (+act) polyclonal stimulation with anti-CD3/CD28 beads, as measured by qPCR and normalised relative to RNU6-2 and SCARNA17.

Having established that dTregs and their released vesicles interacted with and modified BM-DCs, we addressed whether the changes in the phenotype and function of BM-DCs was mediated at least in part by miRNAs originating from Tregs. A microarray screen was performed to identify differentially expressed miRNAs in dTregs and BM-DCs. Of 319 mouse miRNAs (miRBase 18 definition) analysed, 6 were expressed at significantly higher levels in the Tregs compared to the DCs (p < 0.05; fold-change > 1.5). Identified miRNAs included miR-150-5p, miR-182-5p, miR-125b-5p, miR-3096-5p, miR-3096b-5p and miR-96-5p (Fig. 3A and Supplemental Fig. 1) and their differential expression was confirmed by qPCR (Fig. 3B).

Following TCR activation, T cells miRNAs profiles are modified with some miRNAs being downregulated^28,29^ whilst others are increased^29^. To assess how expression of the aforementioned miRNAs profiles were altered following activation, dTregs were stimulated with anti-CD3/CD28 beads for 24 hours and then analysed for miRNA expression by qPCR. We observed that miR-150-5p (p = 0.02) and miR-125b-5p (p = 0.052) were both down-regulated upon stimulation (Fig. 3C) however miR-182-5p was not affected upon stimulation (p = 0.54).

**Figure 4:**
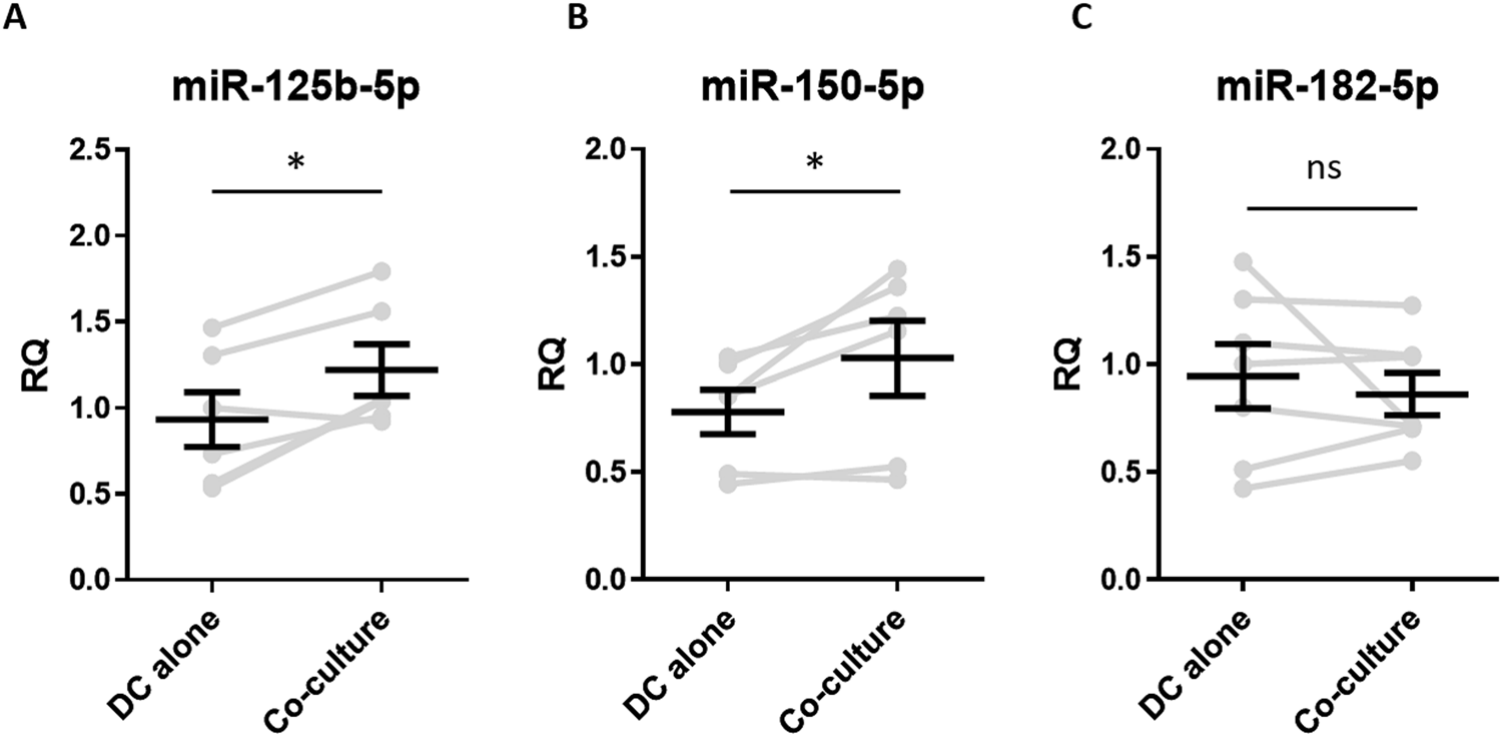
BM-DCs obtain dTreg miRNAs following co-culture with Tregs. Bar graphs showing the relative quantity (RQ) of (**A)** miR-125b-5p, (**B)** miR-150-5p, and (**C)** miR-182-5p expressed by FACS sorted CD11c^+^CD4^−^ DCs BM-DCs following co-culture with dTregs, as measured by qPCR and normalised relative to RNU6-2 and SCARNA17. DC-alone, represents BM-DCs cultured alone and FACS sorted. Data from 6 individual experiments is shown; each grey line represents an individual experiment and the pooled mean ± SEM of these experiments is shown in black. Statistical significance was determined using a two-tailed paired Student’s t-test where *p < 0.05. ns = not significant.

To assess whether intercellular transfer of the aforementioned miRNAs might occur between Tregs and DCs, BALB/c BM-DCs were co-cultured with dTregs at a 1:1 ratio for 24 hours, CD4^-^CD11c^+^ DCs were isolated by FACS sorting (purity > 99%, data not shown) and miRNAs present in the DCs were evaluated by qPCR. DCs co-cultured with dTregs contained significantly more miR-125b-5p (p = 0.022) and miR-150-5p (p = 0.039) compared to DCs cultured alone (Fig. 4A and B, respectively). However, no change in the presence of miR-182-5p (p = 0.54) was observed (Fig. 4C). These observations demonstrate that certain miRNAs, which are highly expressed in Tregs, are present at higher levels in BM-DCs following co-culture, suggesting that intercellular miRNA transfer may have occurred.

### Treg EVs contain specific miRNAs

**Figure 5:**
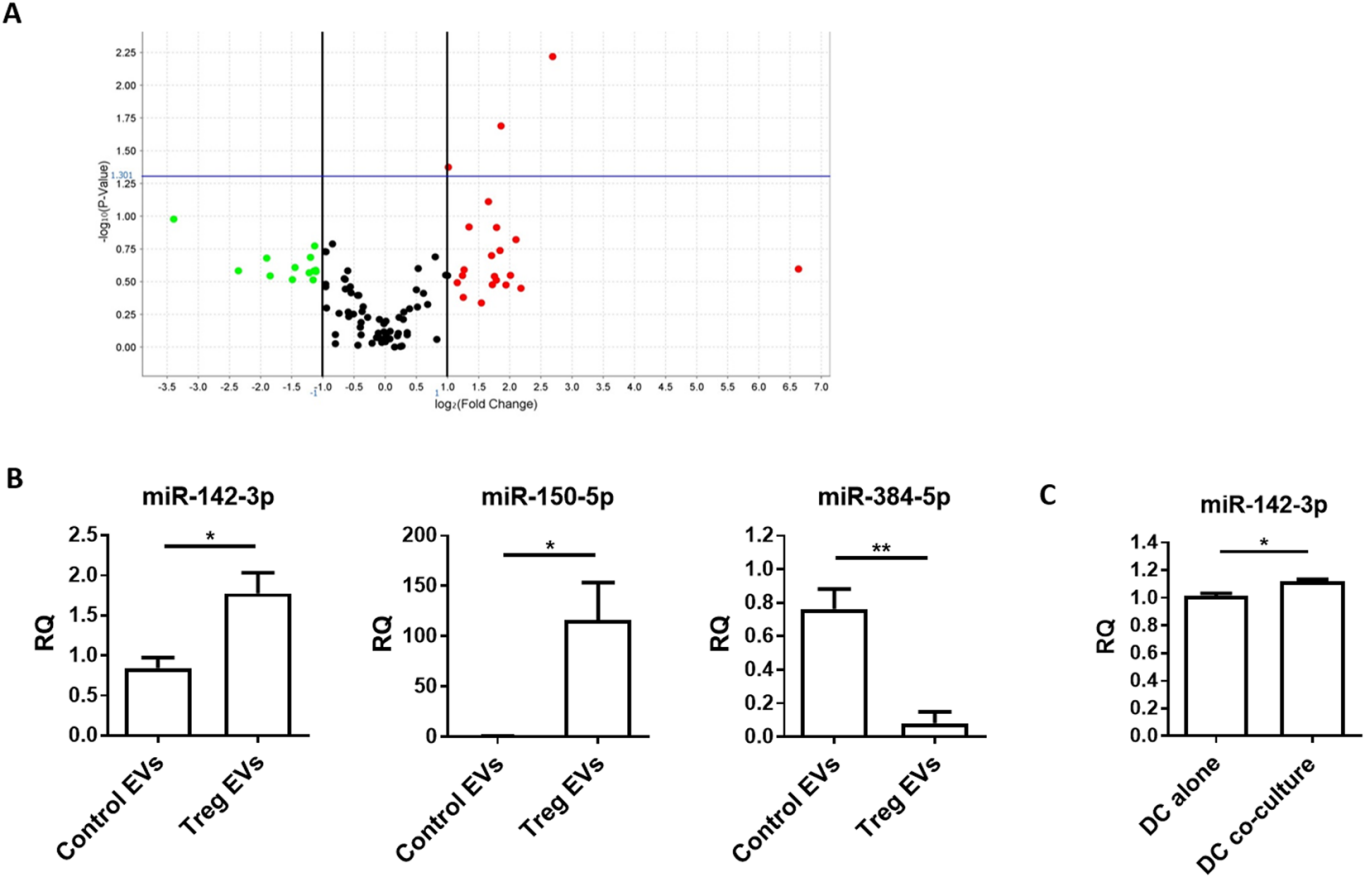
Murine CD4^+^CD25^+^ Treg EVs contain different miRNA compared to control T cell EVs. (**A)** dTreg and control FoxP3^-^ T cell EVs (n = 3 per group) isolated by ExoQuick-TC were lysed and total RNA was purified and assessed using NanoDrop™ spectrophotometer and Agilent 2100 Bioanalyser. EVs miRNA was reverse transcribed into cDNA and pre-amplified before miRNA detection using QuantStudio™ 12 K Flex Real-Time PCR System with the OpenArray® Platform. The volcano plot shows the relation between the p-value and the log fold change between FoxP3^−^ T cell and Treg EV miRNA expression levels. The blue line indicates the inverse log_10_ of the p-value = 0.05. (**B**) Bar graphs (mean + SEM) showing the relative quantity of miR-142-3p, miR-150-5p, and miR-384-5p by control T cell EVs (Control EVs) and Treg EVs (Treg EVs) as measured by qPCR and normalised relative to RNU6-2. Data pooled from 3 individual experiments that were performed in triplicates. Statistical significance was determined using a two-tailed Student’s t-test where *p < 0.05 and **p < 0.01. (**C**) Bar graphs (mean+SEM) showing the relative quantity of miR-142-3p as measured by qPCR and normalised relative to RNU6-2 in FACs sorted CD11c^+^CD4^–^BM-DCs cultured alone (DC alone) or with dTregs (DC co-culture) overnight at a 1:1 ratio. Data pooled from 3 individual experiments that were performed in triplicates. Statistical significance was determined using a two-tailed paired Student’s t-test where *p < 0.05.

To assess whether the increase in miRNAs seen in the BM-DCs following co-culture was due to the delivery of these RNA species via Treg EVs, we analysed the total RNA contents of EVs derived from dTregs and from a control CD4^+^FoxP3^–^ T cell line (Fig. 2A supplemental). As expected, miRNA extracted from EVs contained no ribosomal RNA, as evident by the absence of 18 S and 28 S peaks (Supplemental Fig. 4)^30–32^ whilst conversely, RNA isolated from the two T cell preparations did contain these larger ribosomal RNA species, as seen on the electropherogram (Supplemental Fig. 4). MiRNAs present in the EVs were profiled using a QuantStudio 12 K Flex Real time PCR system (Supplemental Table 1). A Volcano plot (Fig. 5A) was produced to select miRNAs characterized by more than ± 2-fold differences between Treg and control FoxP3^-^ T cell EV content (Log2 (Fold Change) > 1 or <−1) with a log10(p-value) > 1.301. Comparing the miRNAs profiles of EVs derived from dTreg and control T cells, we observed that many miRNAs were common in both EVs (Fig. 5A and Supplemental Table 1). However, several miRNAs were differentially detected (Fig. 5A and Supplemental Table 1) including three identified miRNAs, miR-142-3p, miR-150-5p and miR-384-5p. This was further confirmed by qPCR (Fig. 5B). For example, miR-384-5p was present at significantly higher levels in EVs isolated from the CD4^+^Foxp3^−^ cell line (p = 0.0073) whilst miR-142-3p was present at significantly higher levels in Treg EVs (Fig. 5B, p = 0.0319). Interestingly, miR-150-5p, a miRNA shown previously to be present in EVs isolated from human CD4^+^ T suppressor cells^33^, was also found in EVs isolated from the murine dTreg line used in this study (Fig. 5B, middle panel, p = 0.0381).

As mentioned, we detected higher levels of miR-142-3p in the Treg EVs compared to control EVs. This miRNA was not identified as being significantly differentially expressed (p = 0.0688; fold change = 0.231) between dTregs and BM-DCs in our initial screen. Given these results, we performed a qPCR analysis on RNA extracted from BM-DCs co-cultured with Tregs to assess whether this miRNA was indeed transferred to DCs. We observed a small but significant increase (p = 0.0267) in the presence of this miRNA in the DCs following interaction with dTregs (Fig. 5C). Taken together, our data suggests that Tregs package miRNAs, such as miR-150-5p and miR-142-3p, into EVs and that these may deliver their miRNA contents to DCs.

### miR-150-5p and miR-142-3p presence is increased in DCs following co-culture with Treg EVs

**Figure 6:**
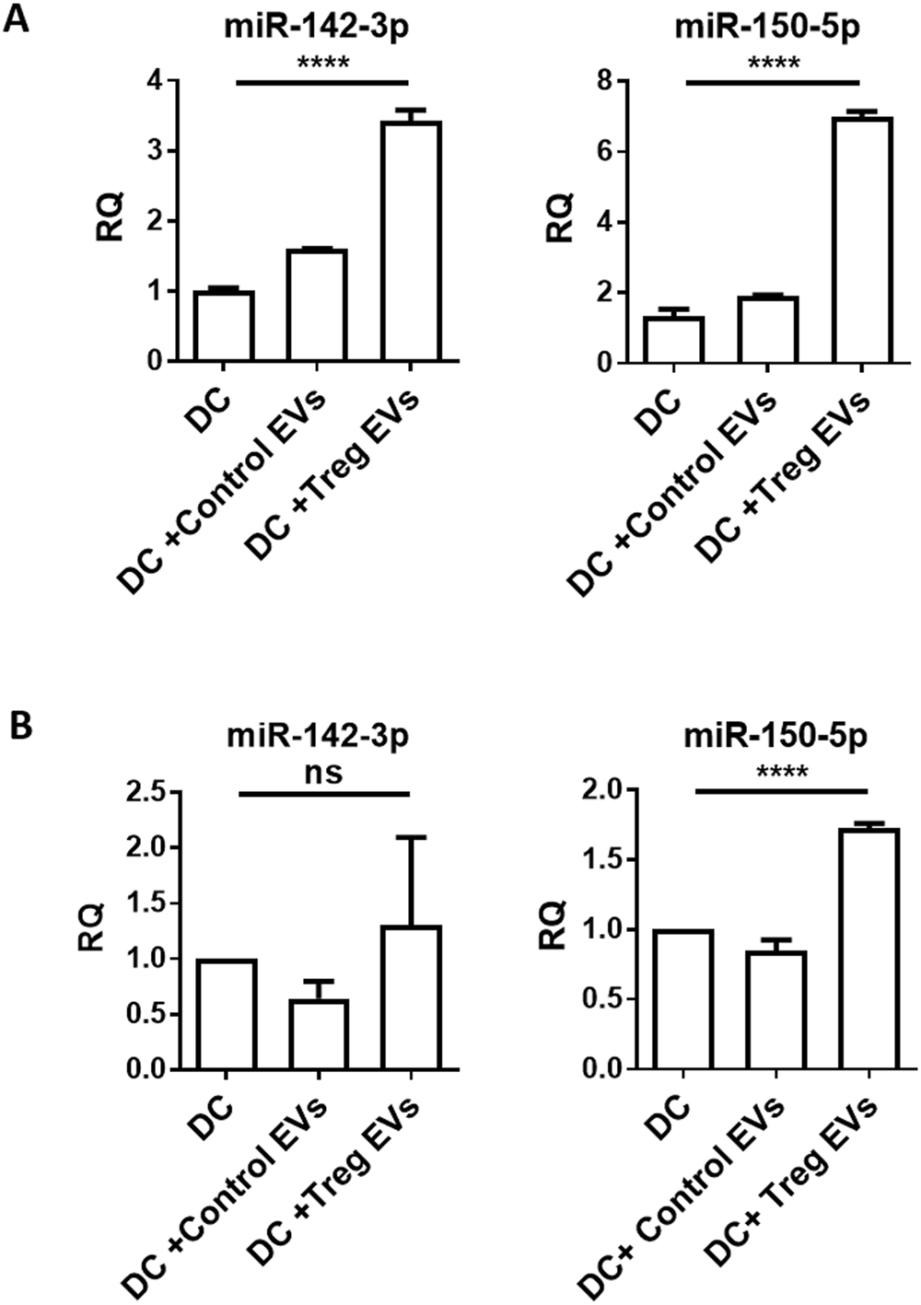
Treg EV derived miRNAs are transferred into DCs. (**A)** Bar graphs show relative quantity (RQ) of miR-142-3p and miR-150-5p in untreated B6 derived DCs (DC), control T cell EV-treated DCs (DC+Control EVs), or Treg EVs-treated DCs (DC+Treg EVs). DCs were treated with EVs for 24 hours, washed thoroughly and miRNA presence was measured by qPCR normalised to RNU6-2. Data represents mean+SEM pooled from 3 individual experiments that were performed in triplicates. Statistical significance was determined using One-way ANOVA and Tukey’s multiple comparison test, ****p < 0.0001. (**B**) Bar graphs show RQ of miR-142-3p and miR-150-5p in untreated Dicer deficient-derived DCs (DC), control T cell EVs-treated Dicer-deficient DCs (DC+Control EVs) or Treg EVs-treated Dicer-deficient DCs (DC+Treg EVs). Dicer-deficient DCs were treated with EVs for 24 hours, washed thoroughly and miRNA presence was measured by qPCR and normalised to RNU6-2. Data shown is mean+SEM representative of 2 experiments that were performed in technical triplicates. Statistical significance was determined using One-way ANOVA and Tukey’s multiple comparison test, ****p < 0.0001 and ns = not significant.

Several miRNAs have been shown to modify cytokine production in DCs, including the two miRNAs previously described^24,34^. In order to assess whether EVs played a role in the intercellular transfer of Treg derived miRNA to DCs, which might help explain the cytokine profiles shown in Fig. 2D, EVs derived from dTregs and the control CD4^+^FoxP3^−^ cells were isolated and co-cultured independently with BM-DCs for 24 hours at a ratio of EVs isolated from 100 donor cells to one recipient cell^35^. After 24 hours, BM-DCs were harvested, washed thoroughly and both miR-150-5p and miR-142-3p presence was measured by qPCR. We observed that both miR-150-5p and miR-142-3p were significantly increased in DCs co-cultured with the Treg derived EVs compared to DCs co-cultured with CD4^+^FoxP3^−^ EVs (Fig. 6A). To further confirm that this was due to the uptake of EVs rather than an induction of these miRNAs in the recipient cell, we utilised Dicer deficient mice as BM-DCs isolated from these mice lack mature miRNAs^19^. Addition of Treg derived EVs, but not EVs isolated from a CD4^+^Foxp3^−^ cell line, to Dicer^−/−^ DCs resulted in the significant increase of miR-150-5p in these cells (Fig. 6B). Although an increase in miR-142-3p within the Dicer deficient DCs was observed this was not significant (Fig. 6B). Overall these observations suggest that miRNAs present in Treg EVs are acquired by BM-DCs and may modulate DC function by affecting the production of cytokines.

## Discussion

The data presented in this manuscript suggests that regulatory T cell derived EVs can modify DCs with respect to the cytokines release following TLR activation. Taken together, the observations that these DCs produce less pro-inflammatory cytokine IL-6 in favour of cytokines such as IL-10 suggest that Treg derived EVs may induce a ‘tolerogenic’ phenotype in DCs. Given that miRNAs play an important role in modulating DC cytokine profiles^24^, we assessed whether dTregs can modulate DC via the intercellular transfer of small RNAs, miRNAs. Human CD4^+^ T cells have been shown to transfer EVs containing miRNA to APCs during immune recognition leading to immune modulation^22^. In agreement with this study, we demonstrated that miRNAs present in murine Treg derived EVs, are acquired by DCs. Indeed, we hypothesise that this intercellular transfer of miRNAs may result in the modification of the cytokine profile seen in treated DCs. Interestingly EV exposure/transfer of miRNAs did not modulate the expression of the co-stimulatory molecule CD80 which was observed following exposure to Treg cells. However many studies have found that over expression of a specific miRNA in BM-DCs did not affect cell surface markers expression while the cytokine profile was affected, so our findings are in line with published data^24^. The functional significance of this is at present unknown. However, we suggest that modification of DCs by EVs will complement/add to the other pathways utilised by Treg cells to induce a ‘tolerant’ APC. However given that several studies have used rat and mouse Treg derived EVs to induce tolerance in the setting of transplantation it is possible that the EVs is one of the major pathways by which Tregs function^36^ through modification of cytokines released by DCs by transferring miRNAs.

Inhibiting pro-inflammatory cytokines production in DCs has been linked to several miRNAs^24^. These include, and are not limited to, miR-21 which inhibits IL-12p35 production, and miR-148/152 which suppress both IL-6 and IL-12 production. miR-21 was significantly expressed in the Tregs used in this study compared to BM-DCs, and this miRNA, as well as miR-148 and miR-152, were identified as present at significant high levels in our Treg EV screen. We however chose to focus on two miRNAs with cytokine modulating properties that were identified as being highly expressed in Treg rather T cell derived EVs; miR-142-3p and miR-150-5p. Ectopic expression of miR-142-3p in human DCs leads to decreased production of IL-12, IL-6 and TNFα after TLR ligation^34,37^. In addition, Sun *et al*. showed that the 3′UTR on IL-6 was a target of miR-142-3p^38^. Transfecting splenic CD11c^+^ murine DCs with pre-miR-142-3p resulted in reduced endogenous levels of IL-6. Conversely knocking down miR-142-3p by transfecting DCs with a miR-142-3p “locked nucleic acid” (LNA) probe led to increased IL-6 production following LPS activation. In these experiments the authors also observed that this miRNA did not modify IL-10 levels following TLR activation^38^.

Recently miR-150-5p was shown in human CD4^+^ T cells to regulate IL-10. Inhibiting miR-150 in primary T cells was correlated with reduced IL-10 production following activation with anti-CD3 and CD46 antibodies^39^. Given these findings we suggest that transferring both miR-150-5p and miR-142-3p may be linked to the increased IL-10 and decreased IL-6 productions seen in DCs treated with Treg derived EVs, respectively. Whether acquisition of other miRNAs, such as miR-21, also present in the Treg derived EVs resulted in production of IL-10 following TLR ligation requires further studies. miR-21 has been shown to indirectly affect IL-10 production. Transfection of pro-miR-21 into RAW264.7 cells led to increased IL-10 production following LPS activation. The authors of this data concluded that LPS induced miR-21 targets PDCA4, a negative regulator of IL-10, leading to increased IL-10 production^40^.

In addition to modulating cytokine production, miR-142-3p has also been shown to attenuate phagocytosis. Overexpression of a miR-142-3p mimic in primary human monocytes and DCs reduced *E coli* fluorescent OVA as well as antibody-mediated phagocytosis, compared to controls^37,41^. Overexpression of miR-142-3p in human DCs has been shown to affect at least 40 genes, some of which are associated with phagocytosis as well as cell signalling associated genes such as PKCα and cell mobility such as N-Wasp^37^. Overexpression of miR-142-3p in dendritic cells has also been linked with decreased T cell activation, which was independent of any changes to MHC or co-receptor expression on the DCs^41^. Overexpression of this miRNA did not affect MHC class II, CD80 or CD86 expression compared to control, non-manipulated, DCs^41^. Given these findings and the observation that miR-142-3p is increased in DCs following exposure to both Tregs and their EVs we suggest that miRNA transfer by Tregs may play a key functional role in their suppressive function with respect to APCs function.

Our flow cytometry observations suggests that Treg derived EVs are acquired by DCs. However, the possibility exists that the EVs do not enter the cell and instead adhere to the surface of the DCs. Several studies have shown that miRNAs present in T cell and Treg derived EVs directly affect validated target molecules present within the recipient cell. For example, c-Myb mRNA, an identified target for miR-150-5p, was downregulated in a miR-150-5p negative hepatocyte derived cellular carcinoma cell line following exposure to human Treg derived EVs containing this miRNA^33^. It is therefore feasible that the miRNAs present in our EVs will affect their mRNA target.

EVs have been shown to contain many other types of RNA species in addition to miRNAs some of which may modify cytokine expression. MiRNAs constitutes only a small fraction of small RNAs found in immune and non-immune cell derived EVs and other species have been identified including, snoRNA, piRNA, lincRNA, rRNA, tRNA^42^ and Y RNA^43^. Indeed snoRNA202 was observed in our dTreg derived EVs. Recently, Y RNA, identified in cardiosphere-derived cell exosomes, was linked to IL-10 expression and secretion^43^. Transfection of macrophages with this Y RNA lead to increased production of this cytokine. Whether other RNA species are present in Treg EVs is out with the scope of this manuscript however given the changes to cytokine expression seen in DCs following treatment with these EVs it is possible that the changes seen are due to the transfer of a combination of RNA species or indeed a combination of miRNAs and cells surface proteins.

We suggest that the intercellular transfer of miRNAs to DCs via EVs is a novel mechanism by which Tregs modify DCs and may represent an important pathway to prevent autoimmunity. Transfer of several miRNA with the potential to induce non-inflammatory cytokine release by DCs may help create a ‘tolerogenic’ environment, although whether the same occurs *in vivo* and how this is modulated in autoimmune conditions has yet to be elucidated. Our findings could provide valuable information regarding the modification of Tregs for therapeutic use. Given that Tregs are now in the clinic^44^, creating Tregs that deliver miRNAs to DCs via EVs may help increase the efficacy of this therapy in patients receiving a transplant or with autoimmune diseases.

## Methods

### Ethics Statement

These studies were approved and conducted in accredited facilities in accordance with The Home Office UK Animals (Scientific Procedures) Act 1986 (Home Office license number PPL 70/7302).

### Mice

Female BALB/c and C57BL/6 (B6) mice were purchased from Harlan UK Ltd. (Biscester, Oxford, UK) and maintained under sterile conditions (Biological Services Unit, New Hunt’s House, King’s College London). Rosa26-ERT2-Cre Dicer floxed/floxed (Dicer knockout mice) were a kind gift from Dr Mark Wilson (Genentech, South San Francisco, California, USA). The Home Office (UK) approved all mouse protocols utilized in this study.

### Flow cytometry

All antibodies were purchased from Thermo Fisher Scientific (Paisley, UK) unless otherwise stated and cells were stained using FACS buffer (PBS containing 5 mM EDTA and 1% FCS). Cells were stained with antibodies specific for the following markers: CD4 (GK1.5), FoxP3 (FJK-16s), CTLA-4 (UC10-4B9), CD73 (eBioTY/11.8), CD11c (N418), H2K^d^ (SF-1-1.1.1), CD80 (16-10A1), CD86 (GL-1), I-A^d^ (39-10-8), CD40 (3/23) (BioLegend) and CD25 (PC61) (BD Biosciences). For intracellular staining, cells were fixed and permeabilised with Foxp3/Transcription Factor Staining Buffer according to manufacturer’s protocols (Thermo Fisher Scientific). Data was acquired on either a BD FACSCalibur™ or BD LSRFortessa™ (BD Biosciences, Franklin Lakes, New Jersey, USA) and analysed using FlowJo 10 software (Tree Star, Ashland, Oregon, USA).

### Bone marrow-derived DC (BM-DC) isolation and culture

Red blood cell-depleted BM cells were incubated with an antibody mixture of YTS-191 (rat anti-mouse CD4), YTS-169 (rat anti-mouse CD8), M5-114 (rat anti-mouse MHC class II) and RA3-3A1 (rat anti-mouse B220) for 30 minutes at 4 °C. Washed cells were then incubated with polyclonal sheep anti-rat IgG coated Dynabeads® (Thermo Fisher Scientific) and antibody labelled cells were removed using a magnet. DC progenitors were cultured in complete media (RPMI-1640, 10% heat-inactivated FCS (General Electric (GE) Healthcare Life Sciences, Buckinghamshire, UK), 100 U/mL penicillin, 100 μg/mL streptomycin, 2 mM L-glutamine, 10 mM HEPES and 50 μM β2-mercaptoethanol (all from Thermo Fisher Scientific) supplemented with GM-CSF at 37 °C/5%CO_2_ for 7 days. Media containing fresh GM-CSF was added on days 2 and 4. At the end of the culture >97% of the cells expressed CD11c (Supplemental Fig. 1). To isolate DCs from Rosa26-ERT2-Cre Dicer floxed/floxed BM an alternative protocol was utilised. Red blood-cell depleted BM cells were cultured in RPMI-1640 with 10% heat-inactivated FCS, 100 U/mL penicillin, 100 μg/mL streptomycin and 2mM L-glutamine supplemented with 200 ng/ml recombinant mouse GM-CSF (Peprotech) at 37 °C in 5% CO2. On days 2, 5 and 7, the media was changed with fresh media (above formulation) with a final concentration of 200 nM of 4-hydrotamoxifen (4-OHT). On day 10, DCs were harvested for experiments and gave a purity of >80%.

### Treg culture, re-stimulation and suppression assay

Experiments were performed using a previously established BALB/c specific Treg line (dTregs)^26^. CD4^+^CD25^+^ T cells were isolated from B6 mice and stimulated once per week by co-culture with irradiated (30 Gy) allogeneic BALB/c DCs at a 1:4 (DC:Treg) ratio in complete media. IL-2 (10 units/ml) was supplemented on days 1, 2 and 4, post re-stimulation. For suppression assays, responder CD4^+^ T cells were isolated from B6 splenocytes using the ‘Dynal® Mouse CD4^+^ Negative Isolation Kit’ (Thermo Fisher Scientific) according to manufacturer’s protocols. APCs were enriched from BALB/c splenocytes using a negative bead selection protocol with YTS-169, YTS-191 and Dynal® beads. Co-cultures contained 2.5 × 10^4^ dTregs, 5 × 10^4^ APCs and 2.5 × 10^4^ CD4^+^ T cells in complete media and were maintained at 37 °C for 72 hours, where 1 µCi ^3^H-thymidine was added in the last 18 hours of culture. Cell proliferation was measured by beta-plate liquid scintillation counting (LKB Wallac 1205 Betaplate® liquid scintillation counter, Austria).

### EV isolation and identification

dTregs maintained in EV-depleted-FCS complete media were incubated overnight in flasks coated with anti-CD3 and anti-CD28 antibodies (5 μg/ml). Culture supernatants were then depleted of cells via centrifugation at 581 × g before being passed through a 0.22 µm filter (Merck Millipore, Billerica, Massachusetts, USA). Small EVs (up to 220 nanometers in size) were isolated from the supernatants using one of two EV isolation methods: ultracentrifugation^20^ and ExoQuick-TC™ (Systems Biosciences, Palo Alto, California, USA) depending on the downstream application. For electron microscopy (EM) visualisation and uptake studies ultracentrifugation was performed whilst for miRNA studies ExoQuick-TC was used. EV isolation via ultracentrifugation purification was performed using a Sorvall Discovery 100 centrifuge with a Sorvall T890 rotor, EVs were pelleted at 100,000 × g at 4 °C for 90 mins, washed with PBS then a further 90 mins spin was performed to re-pellet EVs. ExoQuick-TC™ solution was used according to the manufacturer’s protocol. EVs were suspended in PBS. For identification, EVs were fixed with 4% paraformaldehyde, loaded onto carbon-coated Formwar EM grids for 20 mins. Grids were washed twice with PBS, fixed with 1% glutaraldehyde for 5 minutes. Grids were washed then stained with saturated aqueous uranyl for 10 minutes before analysis using Tecnai T12 BioTWIN electron microscope. Alternatively, a NanoSight LM10 (Nanosight, Malvern, Kk), with kind permission of Dr Sean Davidson (The Hatter Cardiovascular Institute, UK) was used to measure the concentration and size of the particles isolated. Constant flow injection was used and 5 videos of 30 seconds duration were recorded.

### DC and Treg co-culture for confocal microscopy

dTregs were labelled with 1 μM 5(6)-CFDA-SE (Carboxyfluorescein Diacetate, Succinimidyl Ester, CFSE) mixed isomers (ThermoFisher Scientific) and co-cultured with BALB/c DCs at a 1:1 ratio. Real time imaging was performed using a Nikon Eclipse C1 microscope over 1300 seconds at 37 °C.

### DC and Treg co-cultures for miRNA transfer analysis

BALB/c BM-DCs were co-cultured with dTregs at a 1:1 ratio for 18–24 hours. Total cells were harvested, stained with fluorescently conjugated antibodies specific for CD4 and CD11c and CD11c^+^CD4^−^ DCs were isolated by FACS sorting (FACS Aria II, BD). Control DCs cultured in the absence of dTregs were also sorted to nullify the influence of this process on the miRNome of the DCs. Sorted DCs were resuspended in TriZOL (Thermo Fisher Scientific) and stored at −80 °C in preparation for total RNA extraction.

### BM-DC and EV co-culture

4 × 10^7^ dTregs were labelled with 1 μM 5(6)-CFDA-SE (Carboxyfluorescein Diacetate, Succinimidyl Ester, CFSE) mixed isomers (ThermoFisher Scientific) and activated as \described. EVs were isolated using ultracentrifugation and added to 4 × 10^5^ BALB/c derived BM-DCs (day 6) and after 24 hours of co-culture cells were assessed by flow cytometry. To assess cytokine production, Treg EV treated BM-DCs were treated with 100 ng/mL lipopolysaccharides (LPS, Sigma-Aldrich, Germany) for 24 hours and culture supernatants were collected for cytokine analysis. To assess miRNAs, Treg EV treated BM-DCs were harvested, washed twice with PBS and lysed with TRIzol® (Thermo Fisher Scientific) for RNA extraction. Controls were EV untreated DCs.

### RNA, extraction quantification and qPCR

Total RNA was extracted from DCs and Tregs using a phenol/chloroform approach combined with silica-membrane-based RNeasy spin columns (Qiagen, Hilden, Germany). Cells were lysed using TRIzol® and mixed with chloroform. The upper aqueous layer was collected and RNA was isolated using RNeasy spin columns, as per manufacturer’s protocols. Contaminating DNA was removed by treating the eluted RNA with the ‘TURBO DNase Kit’ (Thermo Fisher Scientific) according to manufacturer’ protocols, after which the solution was mixed with phenol-chloroform and the aforementioned process repeated to obtain a pure RNA sample. Total RNA was sent to Exiqon for microarray analysis. Alternatively, complementary DNA (cDNA) was synthesised for qPCR analysis, as detailed below. EVs miRNA was extracted using a SeraMir kit (Systems Biosystems) following the manufacturer’s protocols. Total RNA profile was assessed using an Agilent 2100 Bioanalyser and total RNA nano chips and quantified by NanoDrop™ spectrophotometer (Thermo Fisher Scientific).

cDNA fragments were synthesised using the ‘miScript RT Kit’ (Qiagen, Hilden, Germany), according to manufacturer’s protocols. qPCR reactions were set up with the miScript SYBR Green PCR Kit and primers specific for miR-125b-5p, miR-29a-3p, miR-182-5p, miR-142-3p, miR-150-5p, miR-384-5p, SCARNA17 and RNU6-2, as per manufacturer’s protocols and run on the Applied Biosystems™ ViiA™ 7 Real time PCR system. Data was analysed using the ΔΔC_T_ method where SCARNA17 and RNU6-2 were used as housekeeping controls. The EV miRNA screen was normalised using the global normalisation method with RNU6-2 as an endogenous control.

EV-packaged miRNAs were retrotranscribed and preamplified as previously reported^45^ and were profiled by quantitative polymerase chain reaction (qPCR) on a QuantStudioTM 12 K Flex Real-Time PCR System (Life Technologies, Thermo Fisher Scientific, USA), a platform that simultaneously measures 754 miRNAs (miRBase v14). Global mean was selected as the best normalization method. MiRNA expression was determined using the relative quantification 2-ΔCrt^46^.

### Cytokine measurements

Culture supernatants were analysed using BD™ Cytometric Bead Array Flex Set according to the manufacturer’s protocols to test for mouse TNF, IL-6 and IL-10 cytokines. Beads were acquired on BD LSRFortessa™ and analysed using FCAP Array v3.0.1 software. IL-12 was analysed using an IL-12 ELISA as previously described^47^.

### Statistical analysis

Data represents mean ± standard deviation. Statistical significance was determined using student’s t-tests and one-way ANOVAs with Tukey’s multiple comparisons test. Data was analysed using PRISM software (GraphPad Software, Inc, California, USA).

### Data Availability Statement

The datasets generated during and/or analysed in the current study are available from the corresponding author on reasonable request.

## Electronic supplementary material

Supplemental

## References

[1] Mills KH (2004). Regulatory T cells: friend or foe in immunity to infection?. Nat Rev Immunol.

[2] Safinia N, Leech J, Hernandez-Fuentes M, Lechler R, Lombardi G (2013). Promoting transplantation tolerance; adoptive regulatory T cell therapy. Clin Exp Immunol.

[3] Elinav E, Waks T, Eshhar Z (2008). Redirection of regulatory T cells with predetermined specificity for the treatment of experimental colitis in mice. Gastroenterology.

[4] Safinia N, Scotta C, Vaikunthanathan T, Lechler RI, Lombardi G (2015). Regulatory T Cells: Serious Contenders in the Promise for Immunological Tolerance inTransplantation. Frontiers in immunology.

[5] Shevach EM (2009). Mechanisms of foxp3+ T regulatory cell-mediated suppression. Immunity.

[6] Onishi Y, Fehervari Z, Yamaguchi T, Sakaguchi S (2008). Foxp3+ natural regulatory T cells preferentially form aggregates on dendritic cells *in vitro* and actively inhibit their maturation. Proceedings of the National Academy of Sciences of the United States of America.

[7] Tang Q (2006). Visualizing regulatory T cell control of autoimmune responses in nonobese diabetic mice. Nature immunology.

[8] Yan J, Liu B, Shi Y, Qi H (2017). Class II MHC-independent suppressive adhesion of dendritic cells by regulatory T cells *in vivo*. The Journal of experimental medicine.

[9] Chen J (2017). Strong adhesion by regulatory T cells induces dendritic cell cytoskeletal polarization and contact-dependent lethargy. The Journal of experimental medicine.

[10] Qureshi OS (2011). Trans-endocytosis of CD80 and CD86: a molecular basis for the cell-extrinsic function of CTLA-4. Science.

[11] Chen X, Du Y, Hu Q, Huang Z (2017). Tumor-derived CD4+ CD25+ regulatory T cells inhibit dendritic cells function by CTLA-4. Pathol Res Pract.

[12] Bopp T (2007). Cyclic adenosine monophosphate is a key component of regulatory T cell-mediated suppression. J Exp Med.

[13] Ring S, Karakhanova S, Johnson T, Enk AH, Mahnke K (2010). Gap junctions between regulatory T cells and dendritic cells prevent sensitization of CD8(+) T cells. The Journal of allergy and clinical immunology.

[14] Liang B (2008). Regulatory T cells inhibit dendritic cells by lymphocyte activation gene-3 engagement of MHC class II. J Immunol.

[15] Mellor AL, Munn DH (2004). IDO expression by dendritic cells: tolerance and tryptophan catabolism. Nat Rev Immunol.

[16] Sarris M, Andersen KG, Randow F, Mayr L, Betz AG (2008). Neuropilin-1 expression on regulatory T cells enhances their interactions with dendritic cells during antigen recognition. Immunity.

[17] Mavin E (2017). Human Regulatory T Cells Mediate Transcriptional Modulation of Dendritic Cell Function. Journal of immunology.

[18] Bryniarski K (2013). Antigen-specific, antibody-coated, exosome-like nanovesicles deliver suppressor T-cell microRNA-150 to effector T cells to inhibit contact sensitivity. J Allergy Clin Immunol.

[19] Okoye IS (2014). MicroRNA-Containing T-Regulatory-Cell-Derived Exosomes Suppress Pathogenic T Helper 1 Cells. Immunity.

[20] Smyth LA (2013). CD73 expression on extracellular vesicles derived from CD4+ CD25+ Foxp3+ T cells contributes to their regulatory function. Eur J Immunol.

[21] Kowal J (2016). Proteomic comparison defines novel markers to characterize heterogeneous populations of extracellular vesicle subtypes. Proceedings of the National Academy of Sciences of the United States of America.

[22] Mittelbrunn M (2011). Unidirectional transfer of microRNA-loaded exosomes from T cells to antigen-presenting cells. Nat Commun.

[23] Nazimek K (2015). Macrophages play an essential role in antigen-specific immune suppression mediated by T CD8(+) cell-derived exosomes. Immunology.

[24] Smyth LA, Boardman DA, Tung SL, Lechler R, Lombardi G (2015). MicroRNAs affect dendritic cell function and phenotype. Immunology.

[25] Tsang JY (2009). Indefinite mouse heart allograft survival in recipient treated with CD4(+)CD25(+) regulatory T cells with indirect allospecificity and short term immunosuppression. Transpl Immunol.

[26] Tsang JY (2008). Conferring indirect allospecificity on CD4+ CD25+ Tregs by TCR gene transfer favors transplantation tolerance in mice. J Clin Invest.

[27] Golshayan D (2007). *In vitro*-expanded donor alloantigen-specific CD4+ CD25+ regulatory T cells promote experimental transplantation tolerance. Blood.

[28] Bronevetsky Y (2013). T cell activation induces proteasomal degradation of Argonaute and rapid remodeling of the microRNA repertoire. The Journal of experimental medicine.

[29] Gutierrez-Vazquez C (2017). miRNA profiling during antigen-dependent T cell activation: A role for miR-132-3p. Sci Rep.

[30] Eldh M, Lotvall J, Malmhall C, Ekstrom K (2012). Importance of RNA isolation methods for analysis of exosomal RNA: evaluation of different methods. Mol Immunol.

[31] Lasser, C., Eldh, M. & Lotvall, J. Isolation and characterization of RNA-containing exosomes. *J Vis Exp*, e3037, 10.3791/3037 (2012). 10.3791/3037 PMC3369768 22257828

[32] Crescitelli, R. *et al*.Distinct RNA profiles in subpopulations of extracellular vesicles: apoptotic bodies, microvesicles and exosomes. *Journal of extracellular vesicles* **2**, 10.3402/jev.v2i0.20677 (2013). 10.3402/jev.v2i0.20677 PMC3823106 24223256

[33] Torri A (2017). Extracellular MicroRNA Signature of Human Helper T Cell Subsets in Health and Autoimmunity. The Journal of biological chemistry.

[34] Fordham JB, Naqvi AR, Nares S (2015). Regulation of miR-24, miR-30b, and miR-142-3p during macrophage and dendritic cell differentiation potentiates innate immunity. J Leukoc Biol.

[35] Villarroya-Beltri C, Gutierrez-Vazquez C, Sanchez-Madrid F, Mittelbrunn M (2013). Analysis of microRNA and protein transfer by exosomes during an immune synapse. Methods Mol Biol.

[36] Aiello S (2017). Extracellular vesicles derived from T regulatory cells suppress T cell proliferation and prolong allograft survival. Sci Rep.

[37] Naqvi AR, Fordham JB, Nares S (2015). miR-24, miR-30b, and miR-142-3p regulate phagocytosis in myeloid inflammatory cells. Journal of immunology.

[38] Sun Y (2013). PU.1-dependent transcriptional regulation of miR-142 contributes to its hematopoietic cell-specific expression and modulation of IL-6. Journal of immunology.

[39] King BC (2016). CD46 Activation Regulates miR-150-Mediated Control of GLUT1 Expression and Cytokine Secretion in Human CD4+ T Cells. Journal of immunology.

[40] Sheedy FJ (2010). Negative regulation of TLR4 via targeting of the proinflammatory tumor suppressor PDCD4 by the microRNA miR-21. Nature immunology.

[41] Naqvi AR, Fordham JB, Ganesh B, Nares S (2016). miR-24, miR-30b and miR-142-3p interfere with antigen processing and presentation by primary macrophages and dendritic cells. Sci Rep.

[42] Nolte-‘t Hoen EN (2012). Deep sequencing of RNA from immune cell-derived vesicles uncovers the selective incorporation of small non-coding RNA biotypes with potential regulatory functions. Nucleic Acids Res.

[43] Cambier L (2017). Y RNA fragment in extracellular vesicles confers cardioprotection via modulation of IL-10 expression and secretion. EMBO Mol Med.

[44] Bluestone JA (2015). Type 1 diabetes immunotherapy using polyclonal regulatory T cells. Science translational medicine.

[45] Pergoli L (2017). Extracellular vesicle-packaged miRNA release after short-term exposure to particulate matter is associated with increased coagulation. Particle and fibre toxicology.

[46] Livak KJ, Schmittgen TD (2001). Analysis of relative gene expression data using real-time quantitative PCR and the 2(-Delta Delta C(T)) Method. Methods.

[47] Smyth LA (2013). Tolerogenic Donor-Derived Dendritic Cells Risk Sensitization *In Vivo* owing to Processing and Presentation by Recipient APCs. Journal of immunology.

