# Supplementary material for: Regulatory T cell-derived extracellular vesicles modify dendritic cell function
Source: Sci Rep. 2018 Apr 17;8:6065. doi: 10.1038/s41598-018-24531-8 (PMC5904112; doi:10.1038/s41598-018-24531-8)
Supplement: Supplementary file 1 — Supplemental [file 41598_2018_24531_MOESM1_ESM.pdf]

Supplemental Figures

Regulatory T cell-derived extracellular vesicles modify dendritic cell function

Sim L Tung, Dominic A Boardman, Monica Sen, Marilena Letizia, Qi Peng, Nicole Cianci, Laura Dioni, Leo M Carlin, Robert Lechler, Valentina Bollati, Giovanna Lombardi and Lesley A Smyth

Supplemental Figure 1 | Phenotype of BALB/c BM-DC

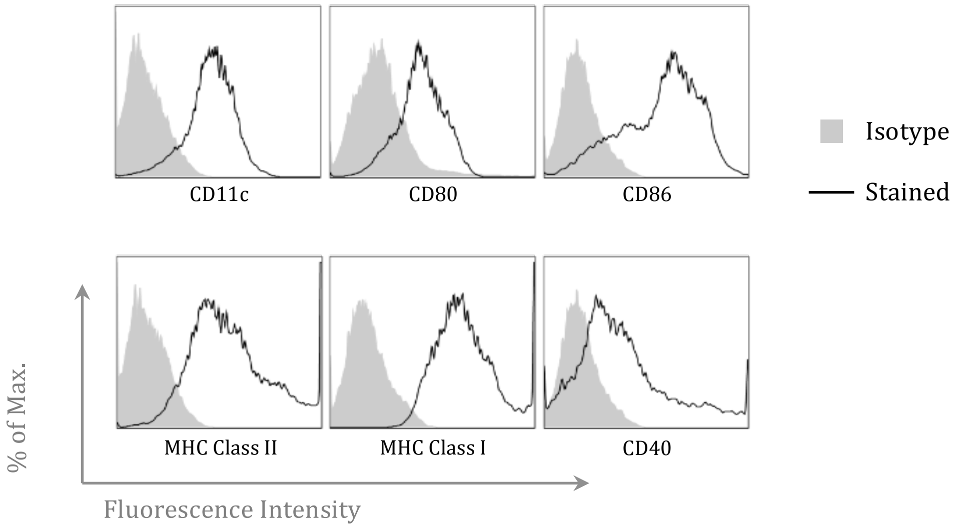

Flow cytometry histogram plots showing the expression for CD11c, CD80, CD86, MHC Class II, MHC Class I and CD40 by viable DCs following staining with fluorescent conjugated antibodies (black line). Isotype control staining is shown in solid grey. Data shown is representative of 6 individual experiments.

Supplemental Figure 2 | Phenotype of CD4^{+}Foxp3^{-} control T cell line

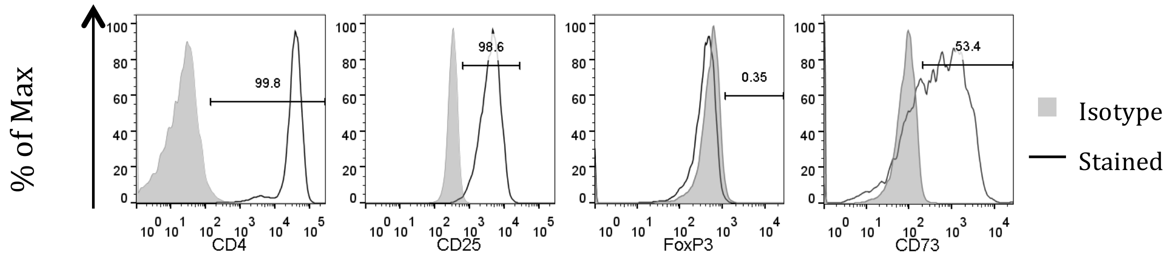

Flow cytometry histogram plots showing the expression levels of CD4, CD25, FoxP3 and CD73 by viable control T cells (black line) following staining with fluorescently conjugated antibodies. Percentage of cells expressing these markers and isotype control staining is shown in solid grey. Data shown is representative of 8 experiments.

Supplemental Figure 3 | Nanoparticle Tracking Analysis (NTA) of Treg-derived EVs

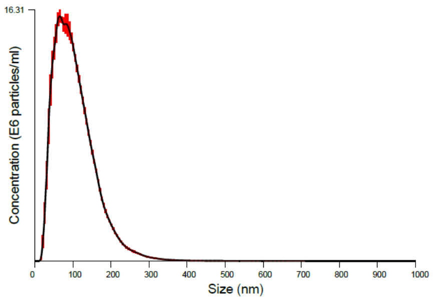

Size Distribution: Mean: 104 +/- 1.1 nm
Mode: 66 +/- 5.9 nm
SD: 53 +/- 0.8 nm

Particle size distribution of Treg derived EVs isolated from culture supernatants by EXoQuick-TC following overnight activation with plate bound

anti-CD3 and anti-CD28 antibodies. Particles were measured by nanoparticle tracking analysis on a LM-10 NanoSight instrument.

Supplemental Figure 4 | Treg EVs contain small RNA species

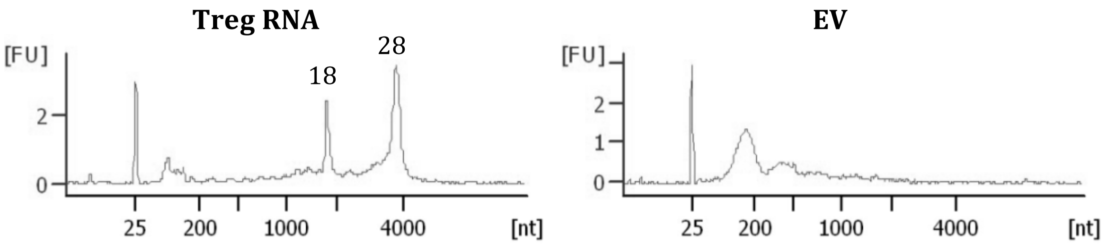

Bioanalyser analysis of RNA isolated from Tregs (left) and Treg derived EVs (right) on a total RNA Nanochip. rRNA peaks are indicated as 18S and 28S subunits in the Treg cells only and are absent in Treg derived EVs. The data is representative of 2 repeats. FU= fluorescence units and nt= nucleotides.

Supplemental Table 1 | Table of the top 40 miRNAs expressed in Treg EVs compared to FoxP3^{-} control EVs

miRNA	Relative Quantity	p-value
miR-150	99.405	0.253
snoRNA202	6.453	0.006
miR-20b	5.306	N/A
miR-2134	4.523	0.355
miR-99b	4.508	N/A
miR-664	4.281	0.151
miR-31	4.212	N/A
miR-34b-3p	4.031	0.283
miR-2138	3.840	0.336
miR-142-3p	3.633	0.020
miR-222	3.589	0.183
miR-203	3.515	N/A
miR-152	3.458	0.122
miR-139-5p	3.443	0.308
miR-221	3.369	0.289
miR-365	3.310	N/A
miR-223-3p	3.300	0.334
miR-223-5p	3.271	0.200
miR-15b	3.152	0.077
miR-362-3p	3.114	N/A
miR-429	2.913	0.461
miR-126	2.540	0.121
miR-342-3p	2.442	N/A
miR-155	2.427	N/A
miR-2182	2.403	0.257
miR-2146	2.386	0.416
miR-331	2.361	0.284
miR-21	2.347	N/A
miR-212	2.230	0.322
miR-340-5p	2.017	0.042
miR-132	1.999	0.285
miR-200c	1.964	0.282
miR-148b	1.912	N/A
miR-29a	1.775	0.872
let-7d	1.747	0.204
miR-125a-5p	1.683	N/A
miR-328	1.604	0.473
miR-1198	1.532	0.388
miR-1897-5p	1.522	N/A
miR-744	1.462	N/A
miR-685	1.443	0.251

A table listing the top 40 miRNAs candidates that have higher expression in Treg EVs compared to control T cell EVs. MiRNAs were ranked according to their relative quantity and associated p-values. N/A = p value of 1.
